# Erastin Reverses ABCB1-Mediated Docetaxel Resistance in Ovarian Cancer

**DOI:** 10.3389/fonc.2019.01398

**Published:** 2019-12-19

**Authors:** Hai-Hong Zhou, Xu Chen, Lu-Ya Cai, Xing-Wei Nan, Jia-Hua Chen, Xiu-Xiu Chen, Yang Yang, Zi-Hao Xing, Meng-Ning Wei, Yao Li, Sheng-Te Wang, Kun Liu, Zhi Shi, Xiao-Jian Yan

**Affiliations:** ^1^Department of Gynecology, The First Affiliated Hospital of Wenzhou Medical University, Wenzhou, China; ^2^Guangdong Provincial Key Laboratory of Bioengineering Medicine, Department of Cell Biology & Institute of Biomedicine, National Engineering Research Center of Genetic Medicine, College of Life Science and Technology, Jinan University, Guangzhou, China; ^3^Center for Uterine Cancer Diagnosis & Therapy Research of Zhejiang Province, Women's Hospital and Institute of Translation Medicine, Zhejiang University School of Medicine, Hangzhou, China

**Keywords:** erastin, docetaxel, ABCB1, ovarian cancer, ferroptosis

## Abstract

Overexpression of drug efflux transport ABCB1 is correlated with multidrug resistance (MDR) among cancer cells. Upregulation of ABCB1 accounts for the recurrence of resistance to docetaxel therapy in ovarian cancer with poor survival. Erastin is a novel and specific small molecule that targets SLC7A11 to induce ferroptosis. In the present research, we explored the synergistic effect of erastin and docetaxel in ovarian cancer. We confirmed that the co-delivery of erastin with docetaxel significantly decreased cell viability, promoted cell apoptosis, and induced cell cycle arrest at G2/M in ovarian cancer cells with ABCB1 overexpression. Mechanistically, erastin dominantly elevated the intracellular ABCB1 substrate levels by restricting the drug-efflux activity of ABCB1 without alteration of the expression of ABCB1. Consequently, erastin can reverse ABCB1-mediated docetaxel resistance in ovarian cancer, revealing that the combination of erastin and docetaxel may potentially offer an effective administration for chemo-resistant patients suffering from ovarian cancers.

## Introduction

Ovarian cancer threatens women's health with high morbidity and mortality and is the leading cause of death in gynecological malignancies (1). The combination of aggressive cytoreductive surgery followed by system chemotherapy is an effective way of treatment (2, 3). Despite how ~80% of patients respond well to such therapies, ~25% of women faced resistant cancer recurrence within 6 months (4). Multidrug resistance (MDR) in ovarian cancer is recognized as the primary cause of failing chemotherapeutic treatments and low survival rates in humans (5). ABCB1 (P-glycoprotein/MDR1), a glycosylated 170-kDa transmembrane protein encoded by the MDR1 gene (6), has emerged as a central drug transport and the best-studied drug transporter in MDR (7). ABCB1 overexpression in cancers contributes to reduced intracellular chemotherapeutics accumulation and brings about resistance against a wide variety of the recently available antineoplastic agents like taxanes (docetaxel), vinca alkaloids (vinblastine), and anthracyclines (doxorubicin) (8–11). Upregulated ABCB1 has been confirmed as the primary protein resulting in MDR in ovarian cancer treated with paclitaxel and related taxane drugs (12–15). Inhibition of ABCB1, therefore, will restore the sensitivity of ABCB1-substrate chemotherapeutic agents, such as docetaxel and doxorubicin. However, drugs under tests that are aimed at ABCB1 to reserve MDR are far from satisfactory for clinical use.

Docetaxel, a new member of the taxane family, has been widely applied in ovarian cancer treatment, especially during first-line chemotherapy in replacement of paclitaxel. It can be delivered alone or together with other chemotherapy drugs, such as carboplatin, to inhibit the microtubule, thus inducing cell cycle arrest (16, 17). Despite the promising anti-cancer effect of docetaxel, nowadays, ovarian cancer has emerged a growing risk of resistance to it. Upregulation of ABCB1 explains part of the resistance mechanism.

Erastin is a small molecule that induces ferroptosis which is a non-apoptotic iron-dependent mode of cell death with smaller mitochondria and increasing membrane density (18). It is the most efficient inhibitor of SLC7A11 at low micromolar concentrations (19). It specifically targets SLC7A11 to prevent cystine import and cause GSH depletion (18, 20). The anticancer property of erastin has been proved to be incredibly effective in a variety of cancers, such as liver cancer, lung cancer, gastric cancer, breast cancer, osteosarcoma, etc. (21–24). Combination therapy of erastin with other chemotherapeutic agents like cisplatin shows a significant synergistic effect in a number of cancers. Nevertheless, whether erastin can enhance the anti-ovarian cancer effect of docetaxel remains unknown. In this study, we revealed that erastin can overcome docetaxel resistance and present as a magical molecule to augment docetaxel efficacy in ovarian cancer by inhibition of ABCB1. This finding may serve as a potential strategy to transform a traditional ferroptosis inducer to elevate therapeutic efficiency for human cancers in the future.

## Materials and Methods

### Cell Culture and Reagents

Human ovarian cancer cells—A2780/Taxol cells—were created by continuous incremental taxol selection in A2780 cells. Cells were grown at 37°C/5% CO_2_ in DMEM. Then, 10% fetal bovine serum (FBS) and antibiotics (100 U/ml penicillin and 100 ng/ml streptomycin) were added. Erastin and ferrostatin-1 were obtained from ApexBio. Deferiprone was purchased from MCE. Docetaxel was bought from Hengrui Medicine. Rhodamine 123 and verapamil were from Sigma-Aldrich. The anti-β-tublin (KM9003T) antibody was purchased from Tianjin Sungene; the Anti-vinculin (bm1611) antibody was obtained from Boster; the anti-PARP (9542) antibody was purchased from Cell Signaling Technologies; the anti-Mcl-1(RLT2679) antibody was from Ruiying; and the Anti-ABCB1 (SC-13131) antibody was purchased from Santa Cruz Biotechnology.

### Cell Viability Assay

Cells were seeded in a 96-well plate, including 6,000 cells, where each well-presented in 100 μl of medium with indicated drugs for 72 h. At the end of the reaction period, 10 μl of 5 mg/ml 3-(4,5-dimethylthiazolyl-2)-2,5-diphenyltetrazolium bromide MTT solution per well was added to the medium for another 4 h. After discarding the whole medium carefully, 100 μl of dimethyl sulfoxide (DMSO) was added to dissolve formazan grains. The absorbance value was read at 570 nm. A Bliss method was used to calculate the IC_50_ (25, 26).

### Apoptosis Assay

Cell apoptosis was detected by a flow cytometry (FCM) assay. In short, cells were washed in cold PBS twice, stained with the binding buffer mixed with Annexin V-FITC and propidium iodide (PI) for more than 15 min in the dark, and then detected by FCM. Fluorescence was valued at an excitation wavelength of 480 nm by 530 and 585 nm filters. The early and late apoptosis rates were measured by FlowJo software (27, 28).

### Cell Cycle Assay

Cells were collected after 48 h of drug incubation and set on cold 70% ethanol for more than 30 min. The ethanol was then discarded and cleaned with cold PBS before staining with PI (50 μg/ml) for 15 min before being measured by FCM with an excitation wavelength of 480 nm through an FL-2 filter (585 nm). ModFit LT 3.0 software (Becton Dickinson) was used to quantify the date (29, 30).

### Western Blot Analysis

Cells were collected and lysed in a RIPA buffer for about 30 min at 4°C. The lysates were then centrifuged at 13,200 × rpm for 10 min to obtain supernatants without cell debris and nuclei. A total of 10% SDS-PAGE gels were used to separate the total proteins and thereby transferred proteins to polyvinylidene difluoride membranes, and 5% BSA was applied to block the membranes for 1 h and then incubated with the indicated primary antibodies overnight. Finally, the proteins were detected by the chemiluminescent detection reagents and a chemstudio plus imaging system (31, 32).

### Rhodamine 123 Accumulation Assay

Cells were treated with or without inhibitors for 1 h before incubation with rhodamine 123 at a dose of 10 μM for an extra 2 h. Verapamil is used as a positive inhibitor of ABCB1. Fluorescent images were taken under fluorescence microscopy. Following that, cells were harvested and cleaned with PBS three times and analyzed with FCM as previously described to measure the fluorescence intensity (33, 34).

### Docking Protocol

Firstly, we obtained the 3D chemical structure of erastin through an online search on PubChem (a national library of medicine). Secondly, we searched the RCSB Protein Data Bank to acquire the human ABCB1 structure, which has been reported to have an active binding site (PDB ID: 6QEX). Then, a docking experiment was executed with Discovery Studio 2.5. The most stable pose with a top-scoring of the ABCB1 complex was selected (35, 36).

### Statistical Analysis

A student's *t*-test was done for date comparison among every group. A *P*-value of <0.05 was considered to indicate statistical significance.

## Results

### ABCB1-Overexpressing Ovarian Cancer Cells Are Resistant to Docetaxel and Erastin

**Figure 1:**
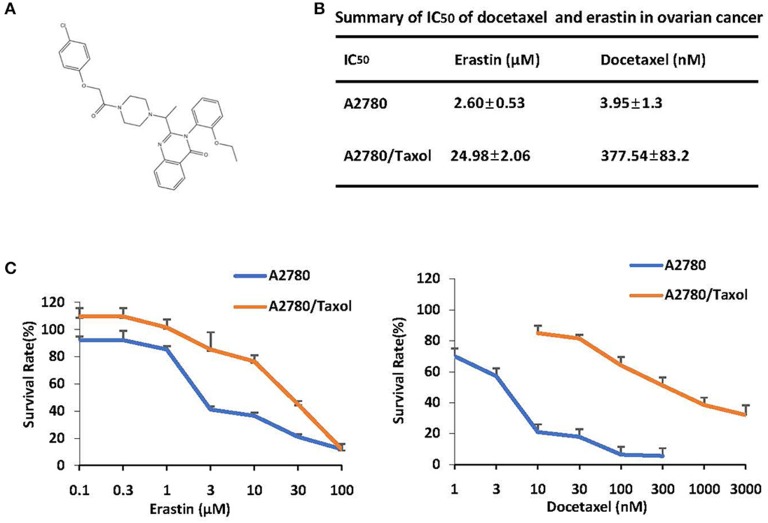
ABCB1-overexpressing ovarian cancer cells are resistant to docetaxel and erastin. **(A)** Chemical structure of erastin download from pubchem. **(B)** Summary of IC_50_ of erastin and docetaxel in the indicated ovarian cancer cells. Cells were grown in 96-well plates for 24 h and treated with the indicated concentrations of erastin or docetaxel for 72 h; cell survival was then determined by an MTT assay. **(C)** The representative growth curves of cells treated with erastin and docetaxel are shown. Data are mean ± SD of three independent experiments.

The structure of erastin was listed in Figure 1A. As shown in Figures 1B,C, the growth rate of ovarian cancer cells is suppressed by docetaxel in a dose-dependent manner. A2780/Taxol, overexpressing the ABCB1 gene, was resistant to docetaxel with the IC_50_ value of 377.54 nM, which was nearly 100-fold higher than A2780 cells. To examine whether erastin induced cytotoxicity in A2780 and A2780/Taxol cells, we exposed cells to increasing concentrations of erastin for 72 h. Consistent with the tolerance toward docetaxel, A2780/Taxol cells possessed more resistance to erastin with the IC_50_ value of 24.98 μM, while A2780 cells were more sensitive to erastin with a lower IC_50_ value of 2.60 nM. Such an interesting co-resistance phenomenon indicated that erastin and docetaxel may share a similar drug-resistance mechanism.

### Erastin Induces Ferroptosis in Ovarian Cancer Cells

**Figure 2:**
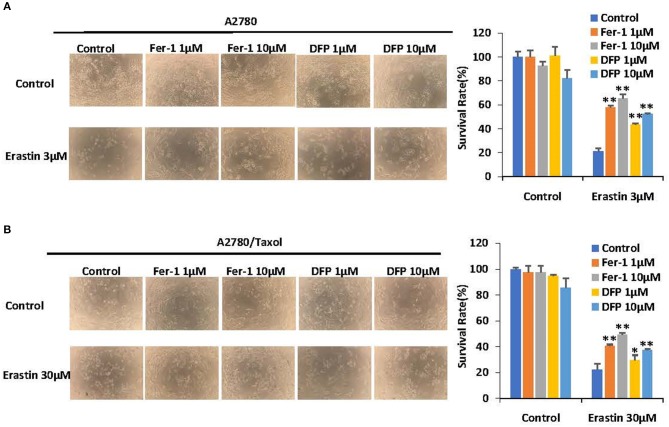
Erastin induces ferroptosis in ovarian cells. A2780 **(A)** and A2780/Taxol **(B)** cells exposed to erastin (3 and 30 μM, respectively) with or without a pretreatment of deferiprone (DFP) or ferrostatin-1 (Fer-1) with the indicated concentrations for 72 h. The morphological changes of cells were observed by microscope as shown. Cell viabilities were analyzed by MTT assay. Data are mean ± SD of three independent experiments. ^*^p < 0.05, ^**^p < 0.01 vs. corresponding control.

To investigate whether erastin induces ferroptosis in ovarian cancer cells, two ferroptosis inhibitors, ferrostatin-1 and deferiprone, were applied to reverse its cytotoxicity. As presented in Figures 2A,B, erastin-induced cell death could be partially reversed by both ferrostatin-1 and deferiprone in both A2780 and A2780/Taxol cells, indicating that erastin can induce ferroptosis in ovarian cancer cells and that there might also be other forms of cell death that can be induced by erastin.

### Erastin Enhances the Sensitivity of Docetaxel in the ABCB1-Overexpressing Ovarian Cancer Cells

**Figure 3:**
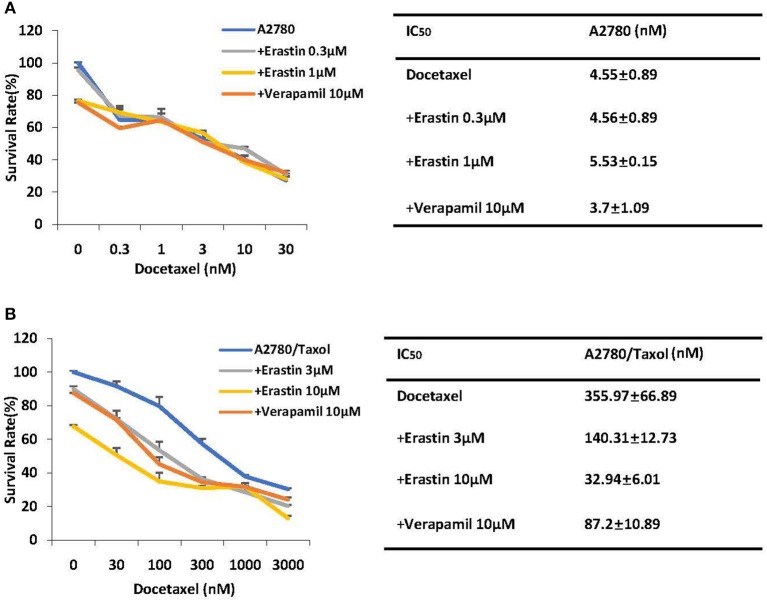
Erastin enhances the sensitivity of docetaxel in the ABCB1-overexpressing ovarian cancer cells. A2780 **(A)** and A2780/Taxol **(B)** cells were treated with the indicated concentrations of docetaxel in combination of erastin or verapamil, respectively, for 72 h. Cell viabilities were analyzed by MTT assay. Data are mean ± SD of three independent experiments. The dose-effect curves and IC_50_ values are shown.

Cancer chemotherapy usually combines drugs for treatment. To explore the combinational effect of erastin and docetaxel in ovarian cancer cells, we co-administrated erastin and docetaxel in both A2780 cells and A2780/Taxol cells. As presented in Figures 3A,B, erastin dose-dependently decreased the IC_50_ values of docetaxel in A2780/Taxol cells, while there was nearly no change in A2780 cells, indicating that erastin can enhance the sensitivity of docetaxel only in the ABCB1-overexpressing ovarian cancer cells.

### Erastin Inhibits the Drug Efflux Activity of ABCB1

**Figure 4:**
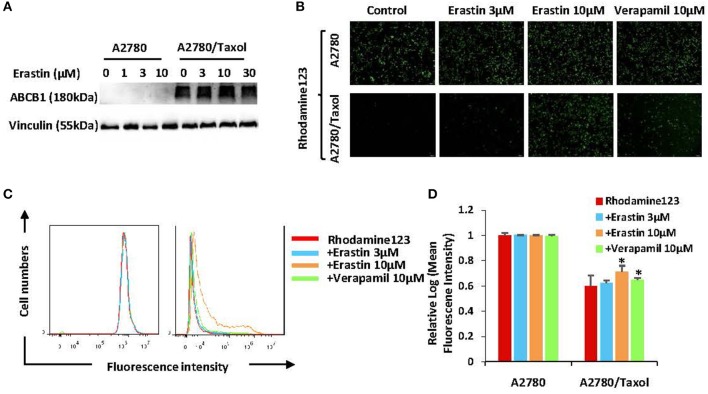
Erastin inhibits the drug efflux activity of ABCB1. A2780 and A2780/Taxol cells were treated with the increasing concentration of erastin for 48 h (0, 1, 3, and 10 as well as 0, 3, 10, and 30 μM, respectively), and the protein expression of ABCB1 was detected by Western blot **(A)**. Cells were incubated with 10 μM rhodamine 123 for another 2 h at 37°C after being pre-treated with the indicated concentrations of erastin and verapamil for 0.5 h at 37°C, as measured by FCM and photographed by fluorescent microscope. The representative graphs **(B)**, charts **(C)**, and quantified data **(D)** are shown. Data are mean ± SD of three independent experiments. ^*^p < 0.05 vs. corresponding control.

To find out whether erastin enhances the sensitivity of docetaxel in the ABCB1-overexpressing ovarian cancer cells is due to downregulation of the expression of ABCB1 or the inhibition of ABCB1 activity, we valued the protein expression of ABCB1 as well as the intracellular aggregation level of rhodamine 123 (ABCB1 substrate) in the pre-incubation of erastin or absence of erastin. The protein expression level of ABCB1 was obviously higher in A2780/Taxol cells than that in A2780 cells (Figure 4A), and erastin did not alter the protein expression of ABCB1 in A2780/Taxol cells. Furthermore, the intracellular rhodamine 123 accumulated in A2780/Taxol cells was at a dramatically lower level compared with A2780 cells, and erastin dose-dependently increased the intracellular rhodamine 123 levels only in A2780/Taxol cells but not in A2780 cells (Figures 4B–D), indicating that erastin can inhibit the drug efflux activity of ABCB1.

### Erastin Enhances Docetaxel-Induced Apoptosis in the ABCB1-Overexpressing Ovarian Cancer Cells

**Figure 5:**
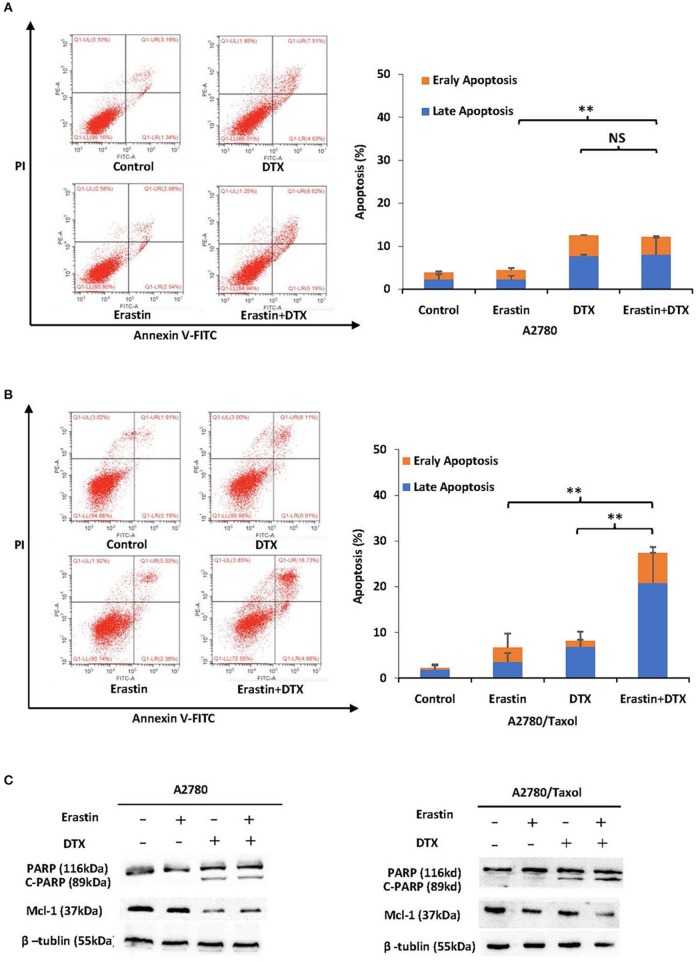
Erastin enhances docetaxel-induced apoptosis in the ABCB1-overexpressing ovarian cancer cells. A2780 **(A)** cells were treated with 1 μM erastin, 10 nM docetaxel alone or in combination for 48 h. A2780/Taxol **(B)** cells were treated with 10 μM erastin and 300 nM docetaxel alone or in combination for 48 h. The apoptosis was detected by FCM with Annexin V/PI staining. The proportions of Annexin V+/PI– and Annexin V+/PI+ cells indicated the early and late stages of apoptosis. The protein expression was examined by Western blot after lysing cells, and β-tublin was used as loading control. The representative charts, quantified results, and Western blot results **(C)** of three independent experiments are shown. DTX, Docetaxel. ^**^p < 0.01 vs. corresponding control.

To further examine the sufficiency of erastin in combination with docetaxel in ovarian cancer cells, cells were incubated under different conditions for 48 h, and the apoptosis rate was detected by FCM. Besides, the related proteins were measured by Western blot. Co-administration of erastin and docetaxel significantly increased the apoptosis rate (both early and late apoptosis) in A2780/Taxol cells but not in A2780 cells (Figures 5A,B). Additionally, as shown in Figure 5C, the co-treatment group showed more increase of cleaved PARP (C-PARP) protein and a higher decrease of Mcl-1 protein than those in either docetaxel or erastin alone group only in A2780/Taxol cells. There was nearly no alteration in A2780 cells, suggesting that erastin can enhance docetaxel-induced apoptosis in the ABCB1-overexpressing ovarian cancer cells.

### Erastin Enhances Docetaxel-Induced Cell Cycle Arrest in the ABCB1-Overexpressing Ovarian Cancer Cells

**Figure 6:**
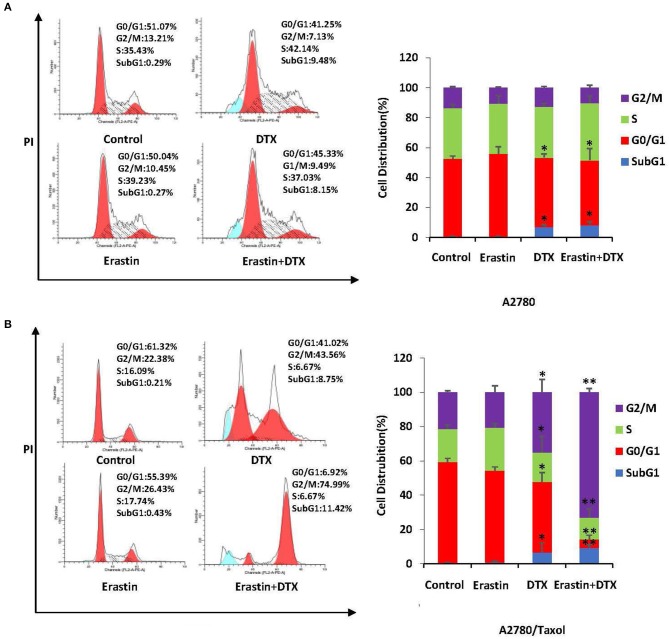
Erastin enhances docetaxel-induced cell cycle arrest in the ABCB1-overexpressing ovarian cancer cells. A2780 **(A)** cells were treated with 1 μM erastin, and 10 nM docetaxel alone or in combination for 48 h. A2780/Taxol **(B)** cells were treated with 10 μM erastin and 300 nM docetaxel alone or in combination for 48 h. The distribution of cell cycle was detected by FCM with PI staining. The representative charts and quantified results of three independent experiments are shown. DTX, Docetaxel. ^*^p < 0.05 and ^**^p < 0.01 vs. corresponding control.

To examine the merging effect of erastin and docetaxel in ovarian cancer cells, the distribution change of cell cycle was measured by FCM with PI staining. As demonstrated in Figures 6A,B, the co-treatment group of erastin and docetaxel dramatically induced more accumulation in the sub-G1 and G2/M phase in comparison with erastin or docetaxel alone treatment only in A2780/Taxol cell but not in A2780 cells, suggesting that erastin can enhance the docetaxel-induced cell cycle arrest in the ABCB1-overexpressing ovarian cancer cells.

### Model for Binding of Erastin to ABCB1

**Figure 7:**
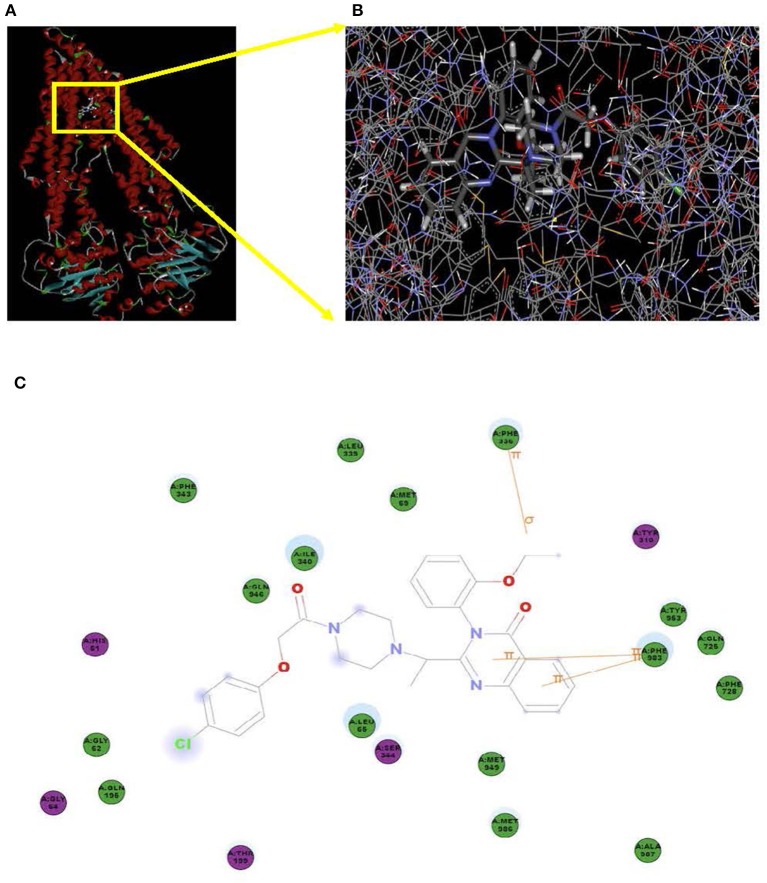
Model for the binding of erastin to ABCB1. The ribbon diagram of 3D structure conformation **(A)** and the optimal predicted binding mode **(B)** of erastin within the human ABCB1 (PDB ID:6qex) binding site are shown. Important amino acids are depicted as lines with the atoms colored (carbon, gray; hydrogen, white; nitrogen, blue; and oxygen, red), and erastin is shown as a ball and stick model with the atoms colored (carbon, gray; hydrogen, white; nitrogen, blue; oxygen, red; and chlorine, green). Yellow lines indicate π-π and π-σ stacking. The dotted green line indicates a hydrogen bonding interaction. The dotted blue line represents the interaction site of erastin and ABCB1 **(C)**.

Docking studies were carried out to demonstrate the binding mechanism of erastin to ABCB1. Firstly, the human ABCB1 crystal structure downloaded online was initially bound with taxol (PDB ID: 6qex). As demonstrated in Figures 7A,B, there existed special interactions between erastin and human ABCB1 (for example, hydrogen bonding), accounting for the stable affinity. The pyridine ring and pyrimidine ring of erastin connected with Phe983 via π-π stacking. The ethyl of erastin interacted with Phe336 through π-σ stacking. The hydron bonds exist between Phe343, Leu339, Met69, Ile340, Gln946, Gly62, Gln195, Leu65, Met949, Met986, Ala987, Phe728, Gln725, Tyr953, and erastin. Meanwhile, the hydrophobic pocket was formed by His61, Gly64, Thr199, Ser344, and TYR310 (Figure 7C), which stabilized the other part of erastin. In short, erastin directly binds to ABCB1 by a special chemical structure connection to suppress the pump activity of ABCB1.

## Discussion

Overexpression of ABCB1 is identified as one of the chief components of cancer chemotherapy failure (13, 37). A recent study declaimed that ABCB1 overexpression is guilty of olaparib resistance (38). Olaparib is a PARP inhibitor that is currently emerging as a promising treatment for ovarian cancer patients with BRCA mutation. Patients with a high expression of ABCB1 may not prospectively benefit from olaparib according to the single BRCA expression context. Therefore, strategies to inhibit ABC transporter proteins are identified as a potentially promising approach to suppress drug efflux in order to overcome drug tolerance. However, we have failed to figure out approved drugs suitable for clinical use for the inhibition of ABCB1, inducing delighted outcomes. A series of trials carried out with third-generation drugs have not been confirmed to have favorable outcomes yet (39). Therefore, developing more effective inhibitors of ABCB1 is urgently required.

Ferroptosis, a newly discovered cell death mode, is characterized by the aberrant accumulation of lipid peroxides in an iron-dependent way (40). Inhibitors of either SLC7A11 or glutathione peroxidase 4 (GPX4) can trigger ferroptosis. Some of the corresponding drugs are erastin and RSL3. Cancer cells often exhibit an increased iron demand to facilitate cell growth (41, 42), indicating that cancer cells may be more vulnerable to ferroptosis. Our work offers data to support the anti-ovarian cancer effect of erastin. Ferroptosis also plays a significant role in the drug resistance of cancer therapy. Firstly, ferroptosis is a non-apoptosis cell death pattern. Faced with the resistance caused by apoptosis-inducing chemotherapy drugs, ferroptotic reagents can serve as a promising strategy in reversing such therapeutic inefficiency. Secondly, persistent drug-tolerant cancer cells are sensitive to ferroptosis (9). In our study, we verified a decrease in the IC_50_ value of docetaxel in the presence of erastin in the ABCB1-overexpressing ovarian cancer cells. The accumulation of rhodamine 123 confirmed that erastin significantly antagonized the drug-efflux function of ABCB1. Moreover, it has been reported that the safety of erastin in xenograft modes is acceptable (43, 44). Inhibition of SLC7A11 by erastin also increased the sensitivity of cisplatin in cancer cells (19, 45), suggesting the combination of erastin and chemotherapeutical reagents can be sufficient for cancer therapy.

In summary, our results demonstrated that erastin could reverse ABCB1-mediated docetaxel resistance in ovarian cancer, suggesting that the combination of erastin and docetaxel may expand the limited options for chemo-resistant ovarian cancers.

## Data Availability Statement

The raw data supporting the conclusions of this article will be made available by the authors, without undue reservation, to any qualified researcher.

## Author Contributions

H-HZ, XC, L-YC, ZS, and X-JY designed the experiments, performed the experiments, analyzed the data, and wrote the paper. X-WN, J-HC, X-XC, YY, Z-HX, M-NW, YL, S-TW, and KL performed the experiments. All authors read and approved the final manuscript.

### Conflict of Interest

The authors declare that the research was conducted in the absence of any commercial or financial relationships that could be construed as a potential conflict of interest.
